# Coil Now, Pipe Later: Two-stage Treatment for Acute Intracranial Aneurysm Rupture

**DOI:** 10.7759/cureus.1876

**Published:** 2017-11-25

**Authors:** Ali S Haider, Tijani Osumah, Hawk Cambron, Suraj Sulhan, Fariha Murshid, Steven Vayalumkal, Richa Thakur, Umair Khan, Kennith F Layton

**Affiliations:** 1 Texas A&M College of Medicine; 2 Department of Urology, Mayo Clinic; 3 School of Medicine, Ross University; 4 Biomedical Engineering, University of Texas at Arlington; 5 School of Medicine, St. George's University; 6 Department of Radiology, Baylor University Medical Center

**Keywords:** endovascular coiling, aneurysm, flow diversion, pipeline embolization device

## Abstract

The two main treatment modalities of acute intracranial aneurysm rupture are endovascular embolization and surgical clipping, each with its own benefits and risks. Endovascular treatment is associated with better outcomes compared to surgical clipping, but is also associated with high recurrence rates. We present the case of a patient with an acutely ruptured intracranial aneurysm, who subsequently underwent partial endovascular coiling acutely, and later underwent flow diversion therapy with the Pipeline Embolization Device. We also review the literature on this topic for further recommendations on treatment options of acute intracranial aneurysm rupture.

## Introduction

Acutely ruptured intracranial aneurysms can pose a treatment challenge in certain clinical situations. Currently, the main two modalities of treatment are endovascular embolization and surgical clipping [1-3]. Endovascular treatment carries the benefit of accessing ruptured aneurysms that are surgically difficult to navigate and have been shown to have a better outcome in patients with subarachnoid hemorrhage (SAH) versus surgical clipping [1,4]. However, endovascular coiling of morphologically complex or large aneurysms is also associated with low rates of angiographic occlusion and high rates of recurrence [5]. The Pipeline Embolization Device (PED) (Medtronic, Irvine, CA), a flow-diverting stent (FDS), has gained momentum in recent years for the treatment of these complex intracranial aneurysms. Yet, its use in the acute ruptured aneurysm setting is limited by the need for dual antiplatelet therapy and the concomitant risk of hemorrhagic complications [1, 5, 6]. Here we present an interesting and unique case of an acutely ruptured irregular intracranial aneurysm that was partially coiled in the acute stage and later underwent definitive treatment with flow diversion.

## Case presentation

**Figure 1:**
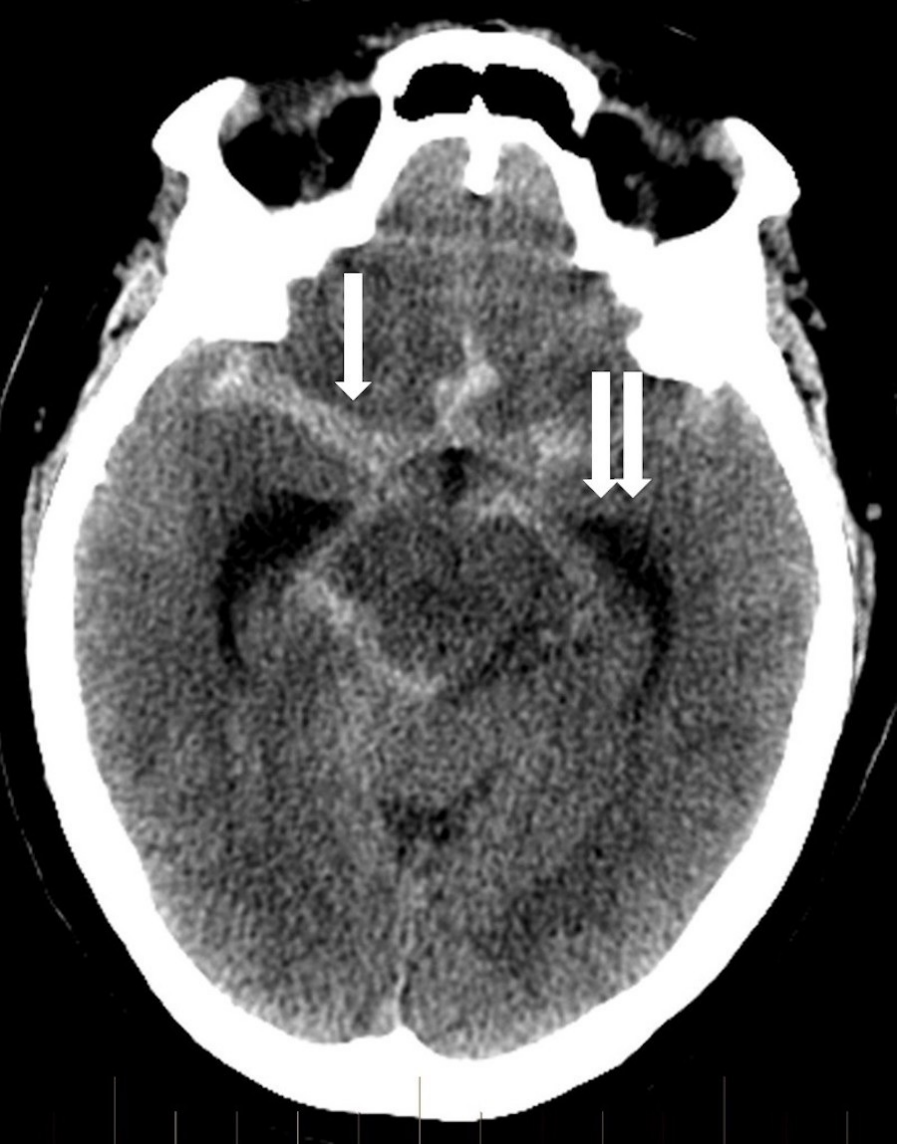
Non-contrast head computed tomography shows diffuse subarachnoid hemorrhage (arrow) and hydrocephalus (double arrow).

A 72-year-old female with a past medical history of hypertension presented to our emergency room with a three-day history of worsening headache complicated by new onset weakness, dizziness and lethargy. She responded easily to verbal commands but was oriented to name only with minimal verbal response. Emergent computerized tomography (CT) of the head showed aneurysmal subarachnoid hemorrhage and developing hydrocephalus (Figure 1).

**Figure 2:**
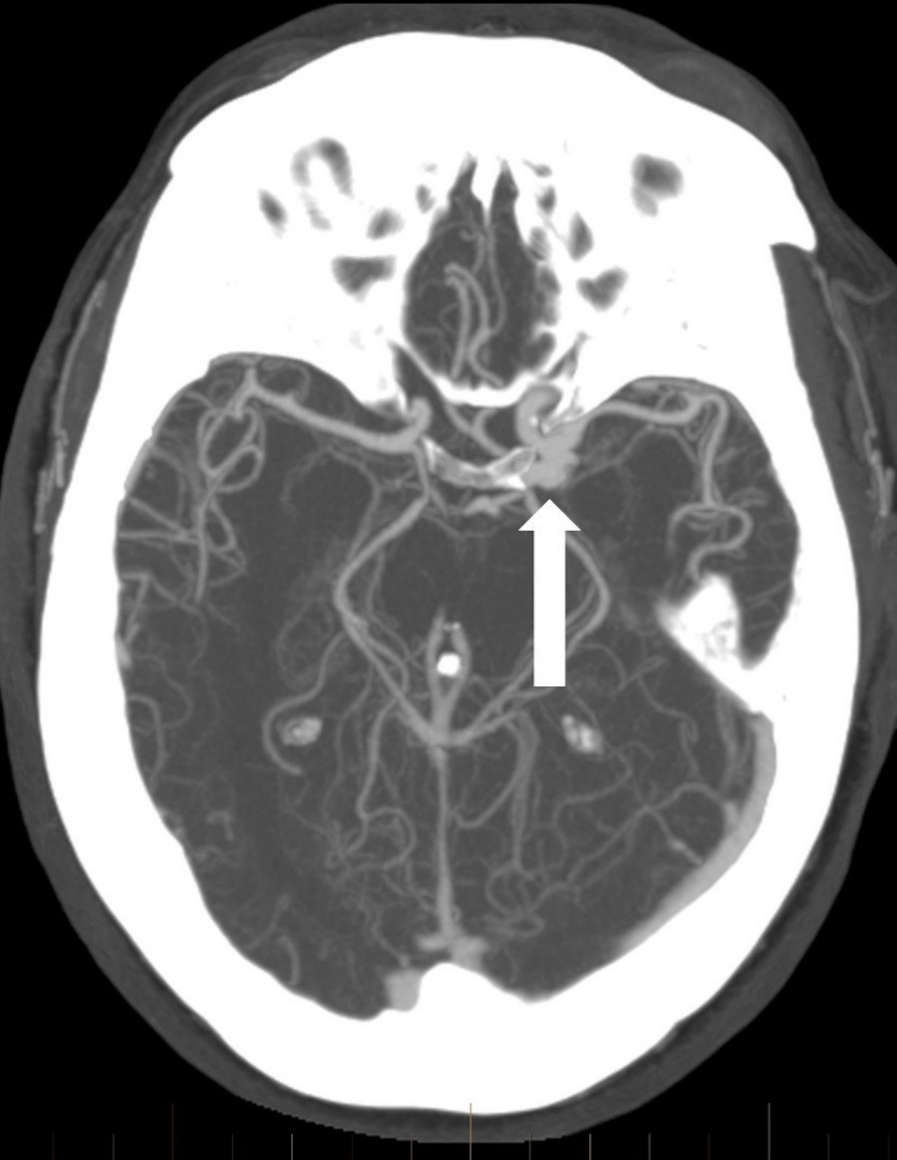
Axial computed tomography angiogram shows the wide neck multilobulated left posterior communicating artery region aneurysm (arrow).

CT angiography revealed a large dysplastic left posterior communicating artery (PCommA) aneurysm with irregular contour, measuring up to 1 cm (Figure 2).

**Figure 3:**
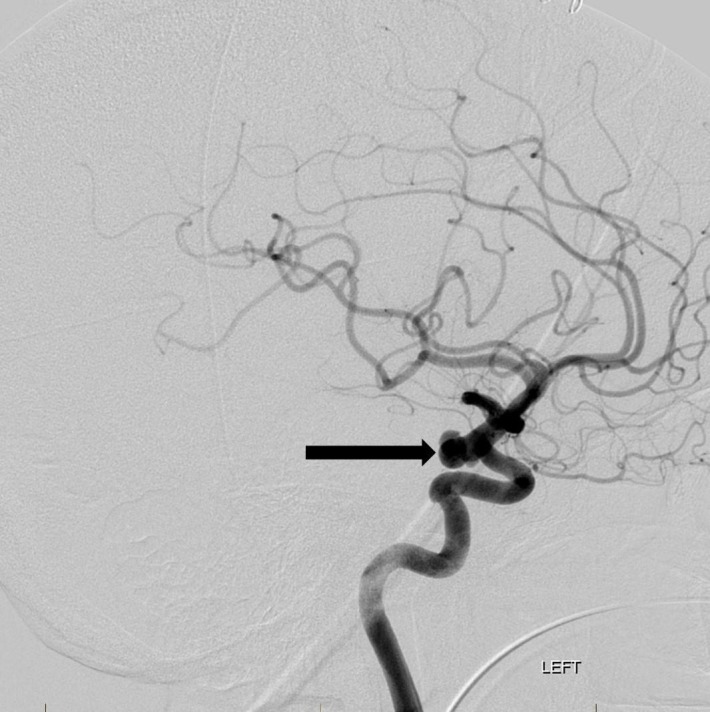
Lateral projection image from a left internal carotid artery angiogram reveals the multilobulated posterior communicating artery region aneurysm along the left posterior carotid wall (arrow). "Left" indicates the patient's left side.

The patient underwent right frontal ventriculostomy placement and was later taken for endovascular embolization of the rupture left PCommA aneurysm. Cerebral angiography confirmed a large, irregular aneurysm with three separate lobules (Figure 3).

**Figure 4:**
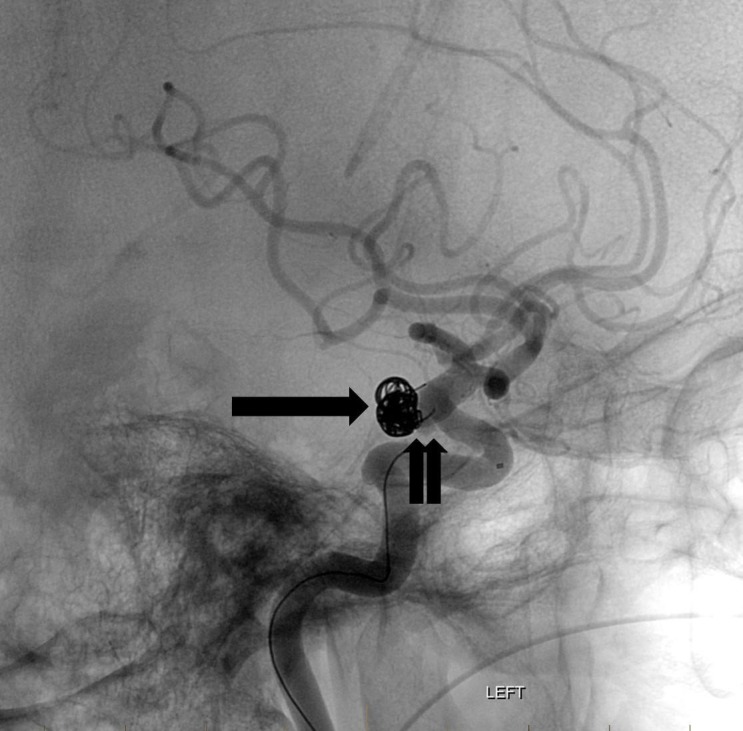
Fluoroscopic lateral projection image after placement of the flow diversion device (double arrow). The coils have a stable configuration within the aneurysm sac (arrow). "Left" indicates the patient's left side.

Two long coils were successfully placed within the body and dome of the two largest lobules but attempts at placing the third coil were unsuccessful as it repeatedly herniated into the parent vessel (Figure 4).

**Figure 5:**
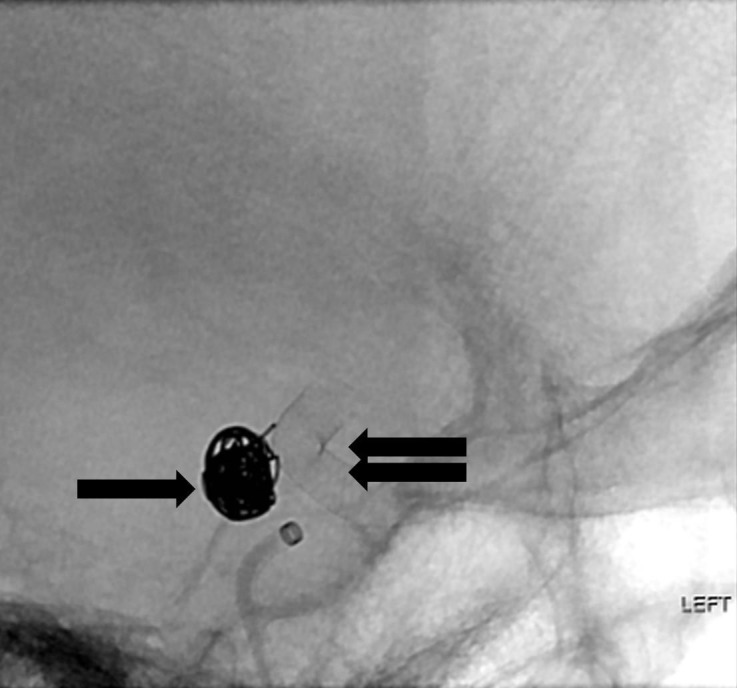
Fluoroscopic lateral projection image after placement of the flow diversion device (double arrow). The coils have a stable configuration within the aneurysm sac (arrow). "Left" indicates the patient's left side.

The decision was made to defer complete embolization until optimal timing of flow diversion therapy. Two weeks later, after the status of the ventriculostomy catheter had been codified, the patient successfully underwent left PCommA pipeline flex embolization device treatment of the aneurysm and was started on aspirin and clopidogrel antiplatelet therapy (Figure 5).

**Figure 6:**
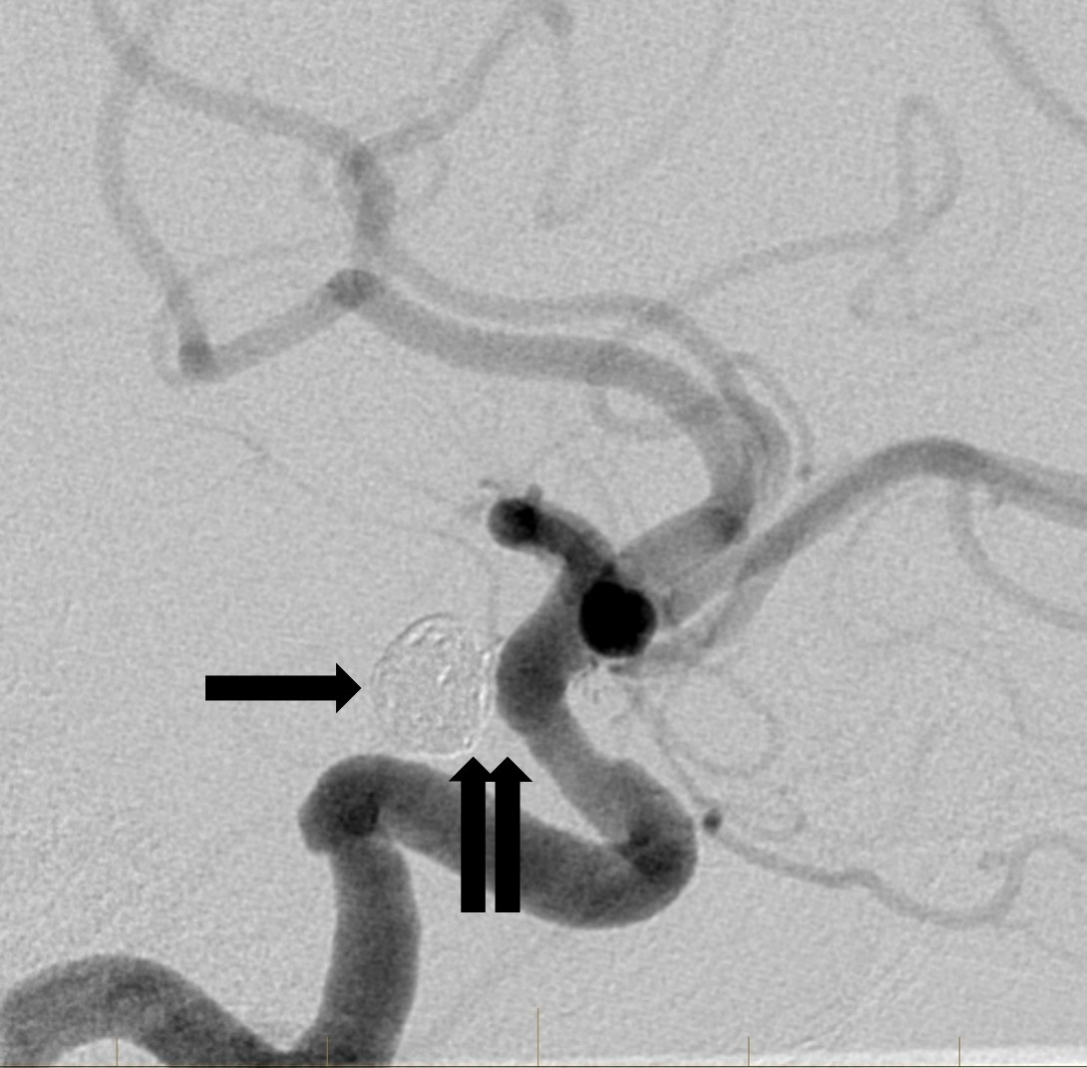
Delayed lateral projection angiographic image from a six-month follow-up study reveals a stable configuration of the coils in the aneurysm sac (arrow). The previously noted area of persistent aneurysm filling at the base of the aneurysm is no longer visible (double arrow). This is consistent with complete aneurysm occlusion.

Follow-up angiogram six months later showed successful occlusion of the aneurysm with no residual aneurysm filling identified (Figure 6).

## Discussion

The PED uses a flexible low-porosity endoluminal sleeve that allows for direct reconstruction of the affected parent artery, reducing the hemodynamic communication between the aneurysm and its parent artery [5, 6]. Over time, this FDS promotes thrombosis within the aneurysm sac, acts as a scaffold for neointima formation, and effectively leads to long-term reconstruction of the parent artery [5]. The need for dual antiplatelet therapy complicates PED use in the acute setting due to the potential risk of spontaneous hemorrhage or necessity of further invasive procedures, such as ventriculostomy placement [7, 8]. Mahaney, et al. described an increased risk of symptomatic intracranial hemorrhage in patients on dual antiplatelet therapy who underwent ventriculoperitoneal shunt placement. Four patients (33%) on dual antiplatelet therapy had a hemorrhagic complication versus zero patients (0%) not on antiplatelet therapy [3]. Few studies have described the novel use of a PED after patients with complex ruptured aneurysms undergo initial staged endovascular coiling in the acute phase where dual antiplatelet therapy is largely contraindicated such as in our patient. In a study by Brinjikji, et al., 31 patients underwent endovascular coiling in the acute phase, 29 of which had experienced aneurysmal rupture, with the intention to possibly undergo flow diversion in the subacute phase. After coiling, 45.2% of their patients developed symptomatic hydrocephalus, subsequently undergoing ventricular drain placement. Additionally, 16.1% of patients had vasospasm that required endovascular intervention, further outlining the potential benefit of avoiding dual antiplatelet use in the acute phase. Given the complexity of the aneurysms, 100% of their patients had incomplete or near-complete occlusion of the aneurysmal sac at the time of flow diversion treatment. Of the 27 patients that underwent subsequent flow diversion treatment, 15 (55.6%) patients had complete occlusion and seven (25.9%) had nearly complete or stable-incomplete occlusions on last imaging studies [1]. In another study, Chalouhi, et al. demonstrated similar success in complex, unruptured aneurysms with 80% of patients achieving complete occlusion, 20% of whom underwent staged coiling followed by PED treatment [7]. Furthermore, the Pipeline for Uncoilable or Failed Aneurysms, a multicenter, prospective single-arm trial, demonstrated PED use in 108 anatomically complicated aneurysms. The mean aneurysm size in this study was 18.2 mm with 20.4% being larger than 25 mm in maximum distension. The study reported technical success in 99.1% of patients who underwent PED placement with major ipsilateral stroke or neurologic death reported in 5.6% of patients [5]. Our case resonates with the studies outlined above to demonstrate how an anatomically complex ruptured intracranial aneurysm may initially be treated with staged endovascular coiling in the acute phase until dual antiplatelet therapy use and FDS treatment is no longer contraindicated. This paradigm allows the aneurysm rupture site to be secured in the short term and the patient to undergo potentially invasive procedures in the acute phase that may not have been possible while on dual antiplatelet therapy.

## Conclusions

Acute intracranial aneurysm rupture is a potentially life-threatening condition requiring prompt treatment. Many treatment modalities are available, each with its own benefits and risks. In the case presented here, a combination of endovascular coiling with subsequent antiplatelet therapy and FDS treatment was utilized to ensure optimal treatment of her anatomically complex intracranial aneurysm. Physicians should be readily prepared to recognize and treat intracranial aneurysm rupture while accounting for the unique anatomical variations of each patient in order to avoid potentially dangerous outcomes.
